# All Trans Retinoic Acid, Transforming Growth Factor β and Prostaglandin E_2_ in Mouse Plasma Synergize with Basophil-Secreted Interleukin-4 to M2 Polarize Murine Macrophages

**DOI:** 10.1371/journal.pone.0168072

**Published:** 2016-12-15

**Authors:** Victor W. Ho, Elyse Hofs, Ingrid Elisia, Vivian Lam, Brian E. Hsu, June Lai, Beryl Luk, Ismael Samudio, Gerald Krystal

**Affiliations:** The Terry Fox Laboratory, British Columbia Cancer Agency, Vancouver, British Columbia, Canada; Universitatsklinikum Freiburg, GERMANY

## Abstract

In previous studies we found that macrophages (MФs) from SH2-containing inositol-5′-phosphatase (SHIP) deficient mice are M2 polarized while their wild type (WT) counterparts are M1 polarized and that this difference in MФ phenotype can be recapitulated during *in vitro* derivation from bone marrow if mouse plasma (MP), but not fetal calf serum, is added to standard M-CSF-containing cultures. In the current study we investigated the mechanism by which MP skews SHIP-/- but not +/+ MФs to an M2 phenotype. Our results suggest that SHIP-/- basophils constitutively secrete higher levels of IL-4 than SHIP+/+ basophils and this higher level of IL-4 is sufficient to skew both SHIP+/+ and SHIP-/- MФs to an M2 phenotype, but only when MP is present to increase the sensitivity of the MФs to this level of IL-4. MP increases the IL-4 sensitivity of both SHIP+/+ and -/- MФs not by increasing cell surface IL-4 or CD36 receptor levels, but by triggering the activation of Erk and Akt and the production of ROS, all of which play a critical role in sensitizing MФs to IL-4-induced M2 skewing. Studies to identify the factor(s) in MP responsible for promoting IL-4-induced M2 skewing suggests that all-trans retinoic acid (ATRA), TGFβ and prostaglandin E_2_ (PGE_2_) all play a role. Taken together, these results indicate that basophil-secreted IL-4 plays an essential role in M2 skewing and that ATRA, TGFβ and PGE_2_ within MP collaborate to dramatically promote M2 skewing by acting directly on MФs to increase their sensitivity to IL-4.

## Introduction

Macrophage (MФ) activation can be broadly divided into M1, pro-inflammatory, and M2, anti-inflammatory subtypes, both of which can alleviate or promote pathologic states [1,2]. For example, M1 MФs, play a critical role in fighting infections but, through their release of cytotoxic oxidizing agents, such as nitric oxide (NO), and inflammatory cytokines, have also been linked to atherosclerotic progression. M2 MФs, on the other hand, through their anti-inflammatory and wound-healing properties, play an essential role in tissue repair and prevention of auto-immune disorders but can promote asthma [3] and cancer progression [4,5]. It has also been demonstrated that MФs can either be immune-activating or immune-suppressing, depending on their activation state, and that reducing M2 MФs and inducing M1 activation improves the prognosis of various cancers in mouse models [4,6–8].

Unlike classically activated M1 MФs, which are typically activated by microbial components like lipopolysaccharide (LPS) and inflammatory cytokines like interferon γ (IFNγ), alternatively activated M2 MФs, which promote humoral and/or anti-inflammatory programs [2,9], are very heterogeneous and their specific phenotype appears to be determined by their cytokine milieu. For example, IL-4-stimulated MФs, currently classified as M(IL-4) MФs [10], have a distinct phenotype from immune-complex plus TLR ligand-stimulated MФs or IL-10 and TGF-β polarized M2 MФs [2,3,9]. Although the growth factors, cytokines, and activating factors responsible for MФ development and polarization have been identified to some extent [1,2,11], much of the cell-cell interactions and support pathways contributing to both homeostatic and inflammatory MФ phenotypes have not been fully elucidated.

In previous studies we showed that the hematopoietic cell-restricted negative regulator of phosphatidylinositol 3-kinase (PI3K), SH2-containing inositol 5′-phosphatase (SHIP, also called SHIP1), represses M2 MФ generation [12]. Specifically, we found that peritoneal and alveolar MФs in SHIP-/- C57BL/6 mice are polarized towards an M2 phenotype, as indicated by high levels of Arg1 and Ym1 expression as well as low IL-12 and high IL-10 production in response to LPS, while their wild type (SHIP+/+) counterparts are M1 skewed [12–14]. Importantly, not only were peritoneal and alveolar MФs more M2 polarized in SHIP-/- than SHIP+/+ C57BL/6 mice but tumor associated MФs (TAMs) were as well, and this correlated with dramatically increased tumor growth rates in SHIP-/- mice [12]. Because of this, we wanted to elucidate the factors responsible for this M2 skewing so that we could prevent/reverse it *in vivo* to slow tumor growth.

Interestingly, *in vitro* culturing of SHIP-/- bone marrow (BM) with M-CSF alone, i.e., standard *in vitro* culture conditions with fetal calf serum (FCS), does not generate M2 MФs, suggesting this M2 skewing is not an intrinsic property of SHIP-/- MФ progenitors [12]. However, we can mimic the *in vivo* differentiation pattern of MФs in SHIP+/+ (i.e., M1) and SHIP-/- (i.e., M2) by adding mouse plasma (MP) to standard *in vitro* cultures [12].

More recent studies from our laboratory have implicated basophils as critical players in M2 and T_H_2 programming in SHIP-/- mice [13,14]. Specifically, we have found that the addition of IL-3 (or GM-CSF to a lesser extent) to BM results in M2 skewing, with resulting SHIP-/- MФs being more M2 skewed than their SHIP+/+ counterparts. Importantly, we found that IL-3 induces this M2 skewing by stimulating basophils and basophil progenitors to survive, proliferate and secrete IL-4 [14].

Taken together, our results to date suggest that both basophils and MP can play a role in the M2 skewing of SHIP+/+ and SHIP-/- MФ progenitors. Herein, we have elucidated the roles that MP, IL-3, basophils and IL-4 play in M2 skewing of SHIP+/+ and -/- MФs and have obtained some insights both into the mechanism by which MP promotes IL-4-induced M2 skewing and the identity of the factors in MP that are responsible.

## Materials and Methods

### Mice

SHIP+/+ and SHIP-/- mice on a mixed C57BL/6 × 129Sv background were generated [15] and housed in specific pathogen-free facilities at the British Columbia Cancer Research Centre. All animal experiments were carried out as described previously [12–14] according to the guidelines for the care and use of animals approved by the University of British Columbia.

### Reagents

Mouse plasma (MP) was obtained, using heparin, from a large pool of mixed C57BL/6 × 129Sv background SHIP+/+ mice and one pooled batch was frozen in aliquots and used for all experiments. The following primary mouse-specific antibodies (Abs) were used for Western blotting: anti-Arg1 and anti-Shc (BD Biosciences, Mississauga, ON, Canada), anti-Ym1 (StemCell Technologies, Vancouver, BC, Canada), anti-SHIP and anti-Grb2 (Santa Cruz, Santa Cruz, CA, USA), anti-GAPDH (Fitzgerald Industries International, Acton, MA, USA), anti-AKT, anti-phospho-AKT (Ser473) (Life Technologies, Burlington, ON, Canada), anti-Erk and anti-phospho-Erk (Thr202/Tyr204) (Cell Signaling Technology, Danvers, MA, USA).

For flow cytometric analyses, recommended Ab/cell ratios were used as outlined in specific data sheets. The following fluorochrome-conjugated Abs were used: anti-CD36-APC (eBioscience, San Diego, CA, USA), anti-IL-4Rα-PE (BD Bioscience) and anti-CD49b-APC (clone DX5) (BioLegend, San Diego, CA, USA). Mouse-specific anti-CD16/CD32 (clone 2.4g2) (BD Biosciences), was used to prevent binding of Abs via their Fc regions to IgG receptors on MФs or other cells. Neutralizing Abs to IL-4 and IL-3 (R&D Systems, Minneapolis, MN, USA) were used in M2-skewing assays with MФs and reagent grade mouse IgG (Sigma-Aldrich, Oakville, ON, Canada) was used as an irrelevant control Ab.

All cytokines were purchased from StemCell Technologies except TGFβ1 (R&D Systems). The following agents tested in M2 skewing assays were purchased from Sigma-Aldrich and stock solutions were prepared as indicated: N-acetylcysteine (NAC) (0.5 M freshly prepared in IMDM), rotenone (1 mM in DMSO), BSO (100 mM freshly prepared in distilled water), DTT (100 mM in distilled water), etomoxir, an inhibitor of fatty acid oxidation (FAO) (100 μM), SB-505124, the TGFβ signaling inhibitor (15 mM in DMSO) and Prostaglandin E_2_ (PGE_2_) (2.5 mM in PBS). The following were also tested: all-trans retinoic acid (ATRA) (10 mM in ETOH) and LY294002 (10 mM in DMSO) from EMD Millipore (Etobicoke, ON, Canada), U0126 (10 mM in DMSO) from Cell Signaling Technology, the COX-2 inhibitors SC-58125 (13 mM in EtOH) from Cayman Chemical (Ann Arbor, MI, USA) and celecoxib (100 mM in DMSO) from LC Laboratories (Woburn, MA, USA) and the ATRA inhibitor (a selective RARα antagonist), BMS195614 (BMS) (10 mM in DMSO) from Tocris Biosciences (Minneapolis, MN, USA). Mouse-specific IL-4 ELISA kits were purchased from BD Biosciences.

### Western analysis

To determine protein concentrations, samples were first solubilized in NP-40 lysis buffer and protein levels assessed using a BCA protein assay kit (Thermo Fisher, Ottawa, ON). Cell lysates, supplemented with concentrated SDS sample buffer to give 1 x SDS sample buffer were boiled for 5 min and subjected to Western analysis as described previously [16].

### Derivation of bone marrow macrophages (progenitor assay)

BM was flushed from tibias and femurs, suspended at 25 mL/mouse BM in MФ medium, i.e., IMDM + 10% fetal calf serum (FCS) (Hyclone; South Logan, UT, USA) + 150 μM monothioglycerol (Sigma-Aldrich) + 100 U/mL penicillin + 100 μg/mL streptomycin (P/S), seeded into tissue culture flasks (75cm^2^) and cultured at 37°C for 3–18 h. Non-adherent BM progenitors were then harvested and mature BMMФs derived by culturing 0.5 x 10^6^ cells/mL in MФ medium with 5–10 ng/mL macrophage colony stimulating factor (M-CSF)(Stemcell Technologies Inc) +/- 5% MP for 6–14 days with medium changes every 3–4 days, which involved spinning down non-adherent cells and reincorporating them back into the culture as necessary.

### Mature macrophage skewing assay

After deriving the MФs for 6–14 days as described above in large tissue culture flasks (175 cm^2^), the medium, along with any remaining suspended cells, was aspirated and discarded. To detach the mature MФs, 10 mL of Accutase (Stemcell Technologies Inc) was added to the flask and incubated for 10 min at 23°C with agitation. Detached MФs were pelleted (300 x g) and resuspended to the desired concentration in MФ medium containing 5–10 ng/ml M-CSF and 200,000 to 400,000 cells were seeded per well into 12 well plates. The macrophages were then treated with the desired skewing agents and/or inhibitors.

For transwell studies, transwell plates (0.4 μm membrane pore size) were purchased from Corning (Lowell, MA, USA). The ratio of basophils (DX5+ cells) (in the top insert) to basophil-depleted progenitors (DX5- progenitors) (bottom well) was 1:10 for all assays.

### Harvesting of macrophages

MФs were harvested using cell dissociation buffer (CDB) (Life Technologies). Before CDB treatment, the medium was aspirated and adherent cells washed with MФ medium to remove non-adherent cells. After 3–5 min of incubation at 23°C, cell flasks or dishes were agitated to obtain single-cell suspensions. Cells were lysed directly in SDS-lysis buffer or with 0.5% NP40 (EMD Millipore) for protein quantification before SDS lysis.

### Peritoneal macrophage isolation

MФ medium (5 ml) was injected a total of 3 times into and aspirated out of the peritoneal cavity of a euthanized mouse. MФs were counted with a hemocytometer, discriminating them from other cells by size and morphology. To adherence select for peritoneal MФs, cells were plated at 0.5–1.0 x 10^6^ cells/mL for 3 h– 16 h in MФ medium, after which the wells were washed twice to remove non-adherent cells.

### Arginase assay

MФs were washed with PBS and lysed with arginase lysis buffer (a 1:1 mixture of 0.1% Triton X-100 (Sigma-Aldrich) and 25 mM Tris-HCl, pH 8.0). Using 100 μL of cell lysate, or a dilution of the lysate up to 100 μL with the same buffer, arginase activity was determined by a colorimetric enzyme assay as described by Morrison and Correll [17]. Briefly, each sample was heat-activated at 55°C for 10 min with 10 μL of 10 mM MnCl_2_. Then, 100 μL 0.5 M L-arginine (pH 9.8) (Sigma-Aldrich) was added and incubated at 37°C for 1 h. The reaction was stopped using 800 μL of a 1:3:7 H_2_SO_4_:H_3_PO_4_:H_2_O (Fisher). The level of urea was measured by adding 40 μL 9% α-isonitrosopropiophenone (Sigma-Aldrich) (w/v in ethanol (ETOH)) and heating at 100°C for 30 min. Urea concentration was determined by reading the absorbance at 550 nm and comparing it to a urea standard curve. Arginase activity was expressed as μg urea/μg protein/h. Protein concentration was determined using the Bradford Assay (BioRad; Mississauga, ON, Canada).

### Basophil isolation and culture

Mature basophils were enriched from BM using EasySep (StemCell Technologies) and APC-conjugated DX5 Ab at 2–5 μg/mL (BioLegend; San Diego, CA, USA) to positively select for CD49b+ cells. Basophil function was assessed at a cell concentration of 2–5 x 10^4^ cells/100 μL in MФ medium, unless otherwise stated.

### Charcoal stripping, lipid extraction and M-CSF depletion

For charcoal stripping, activated charcoal was added to MP at a concentration of 2g/100mL. The mixture was incubated overnight at 4°C on a rotator. The charcoal was pelleted by centrifugation and the plasma supernatant was collected and filter sterilized.

For lipid extraction the procedure of Cham & Knowles was used [18]. Briefly, two volumes of butanol/di-isopropyl ether (40:60, v/v) were added to one volume of MP containing 0.5 mg/mL EDTA and rotated at 23°C for 30 min. The mixture was centrifuged at 2000 rpm for 5 min in a Beckman Coulter Allegra X-12R centrifuge and the aqueous phase removed with a needle and syringe and then washed one time with 2 volumes of di-isopropyl ether. Both the delipidated aqueous phase and the solvent phase containing the lipid fraction were left to evaporate overnight to remove any residual solvent. The next day the dried lipid fraction was re-suspended in methanol and speed-vacuumed for 1 h and re-suspended in methanol as a 10x.

### Statistical analysis

Statistical analyses were performed between SHIP+/+ and -/- samples using the Student’s *t*-test. As well, data presented in bar graphs were tested using both the student’s t-test and the non-parametric Mann Whitney rank test. Both tests yielded the same results in terms of significance. Thus significant results were obtained in these studies regardless of whether normal distribution was assumed. A confidence level of p < 0.05 was considered significant. Results shown are representative of a minimum of 3 experiments, containing 3 replicates/experiment, unless otherwise stated.

## Results

### MP-induced M2 polarization of SHIP-/- MФs involves basophils and IL-4 but not IL-3

**Fig 1:**
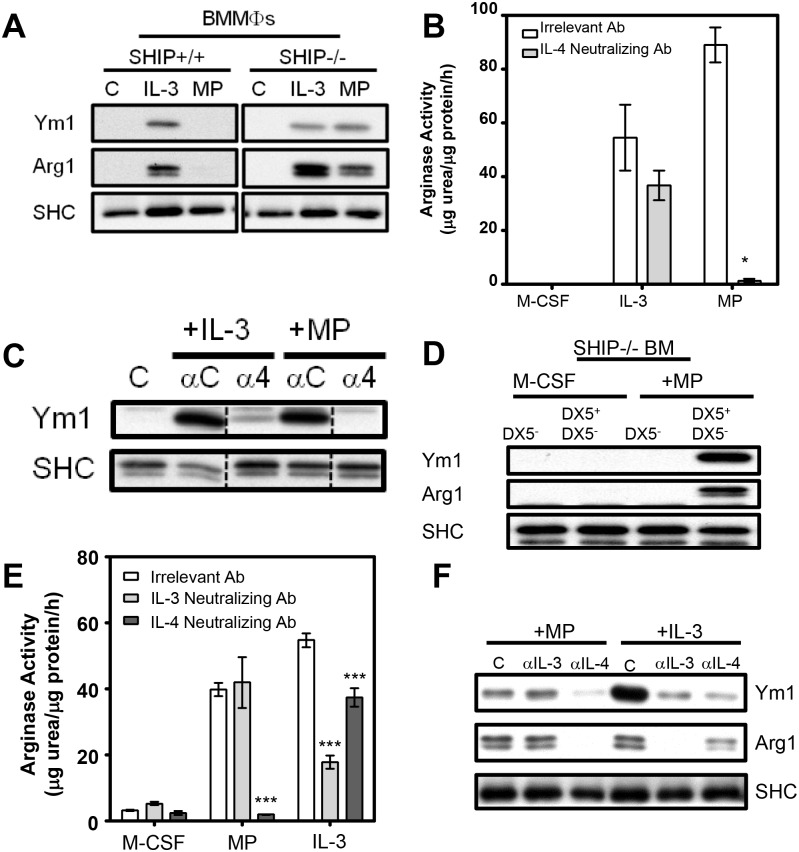
MP-induced M2 skewing of SHIP-/- BM progenitors requires IL-4 and basophils but not IL-3. **A)** Western blots of MФs derived from BM for 6 days with M-CSF ± IL-3 (10 ng/mL) or MP (5%). **B)** Arginase activity of MФs from SHIP-/- BM derived for 6 days with 10 ng/mL M-CSF ± IL-3 (10 ng/mL) or MP (5%) with 2.5 μg/mL of either an irrelevant or IL-4-neutralizing Ab. Data (mean ± SD) are representative of 3 independent experiments performed in triplicate. * p < 0.05 compared to all other conditions. **C)** Western blot of SHIP-/- MФs from (B) probed for Ym1, using SHC as a loading control. Dashed lines indicate where irrelevant lanes have been cropped out. All lanes are part of the same time-exposed film on the same gel. **D)** Western blot of SHIP-/- basophil-depleted (DX5^-^) or basophils (DX5^+^) + DX5^-^ BM derived with M-CSF ± MP (5%). **E)** Arginase activity of SHIP-/- MФs derived from BM for 6 days with M-CSF ± MP (5%) or IL-3 (10 ng/mL) ± neutralizing Ab to IL-3 (2.5 μg/mL) or IL-4 (2.5 μg/mL). **F)** Western blot of cells corresponding to panel (E). *** p < 0.001 compared to relevant control.

In earlier studies we showed that MФs isolated from the peritoneal cavity or lungs of SHIP-/- C57BL/6 mice are M2 skewed (i.e. express both Arg1 and Ym1 and produce low IL-12 and high IL-10 following LPS stimulation) while WT littermate MФs are not [12]. However, we could not mimic this *in vivo* generated difference with standard *in vitro* BM cultures (i.e., with M-CSF + 10% FCS) (Fig 1A) or with IL-3, which we found skews both SHIP+/+ and -/- progenitors to an M2 phenotype (Fig 1A). On the other hand, we could recapitulate our *in vivo* findings if we added mouse plasma (5% MP) to our *in vitro* BM cultures (Fig 1A and [12]). We also found in earlier studies that IL-3 skews both SHIP+/+ and -/- BM progenitors to an M2 phenotype via its stimulation of IL-4 from basophils [13,14]. To determine if MP-induced M2 skewing of SHIP-/- MФ progenitors was dependent on IL4, we added a neutralizing anti-IL-4 Ab to both MP- and IL-3-treated SHIP-/- BMMФ cultures. As expected, given our earlier results [14], the neutralizing Ab to IL-4 modestly reduced IL-3-induced M2-skewing. However, it reduced MP-induced M2-skewing to a far greater extent, as indicated by a more profound reduction in arginase activity (Fig 1B) and Ym1 protein expression (Fig 1C), confirming the involvement of IL-4 in MP-induced M2 skewing.

To look at the role of basophils in MP-induced M2 skewing, SHIP-/- BM was depleted of basophils using the monoclonal Ab, DX5, specific for the cell surface marker CD49b, expressed on basophils. As shown in Fig 1D, basophil-depleted (i.e. DX5- SHIP-/- BM progenitors were incapable of being skewed to an M2 MФ phenotype with MP unless they were reconstituted with basophils (DX5+ cells). Similarly, SHIP+/+ or -/- DX5- BM was incapable of being skewed to an M2 MФ phenotype with IL-3 unless they were reconstituted with basophils (data not shown). This is consistent with our earlier finding that only DX5+ BM cells secrete IL-4 in response to IL-3 [13]. While the use of anti-FcεRI, in conjunction with DX5, would give a more highly purified population of basophils, we did not want to risk FcεRI-mediated activation through the use of an Ab, such as MAR-1, because that would elicit IL-4 secretion, which would confound our results [19,20]. Instead, we used DX5 alone to purify basophils even though it is also expressed on NKT and memory T cells in BM [21,22]. However, preliminary studies in which we enriched these T cells subsets, using anti-CD4, from SHIP-/- BM revealed that they did not produce detectable IL-4 in response to IL-3 or MP (data not shown). We therefore conclude that the DX5+ cells contributing to IL-4 production are, indeed, basophils.

We next asked if MP-induced M2-skewing was dependent on IL-3, using a neutralizing Ab to IL-3 and found that while this anti-IL-3 Ab markedly reduced IL-3-induced M2 skewing, as expected, it had no effect on MP-induced M2 skewing. This was shown via arginase activity measurements (Fig 1E) and Western blotting with Abs to Arg1 and Ym1 proteins (Fig 1F). Thus, MP induces M2 skewing of SHIP-/- BM via an IL-4 but not an IL-3 mediated process.

### MP acts directly on MФs to markedly increase their sensitivity to IL-4-induced M2 polarization

**Fig 2:**
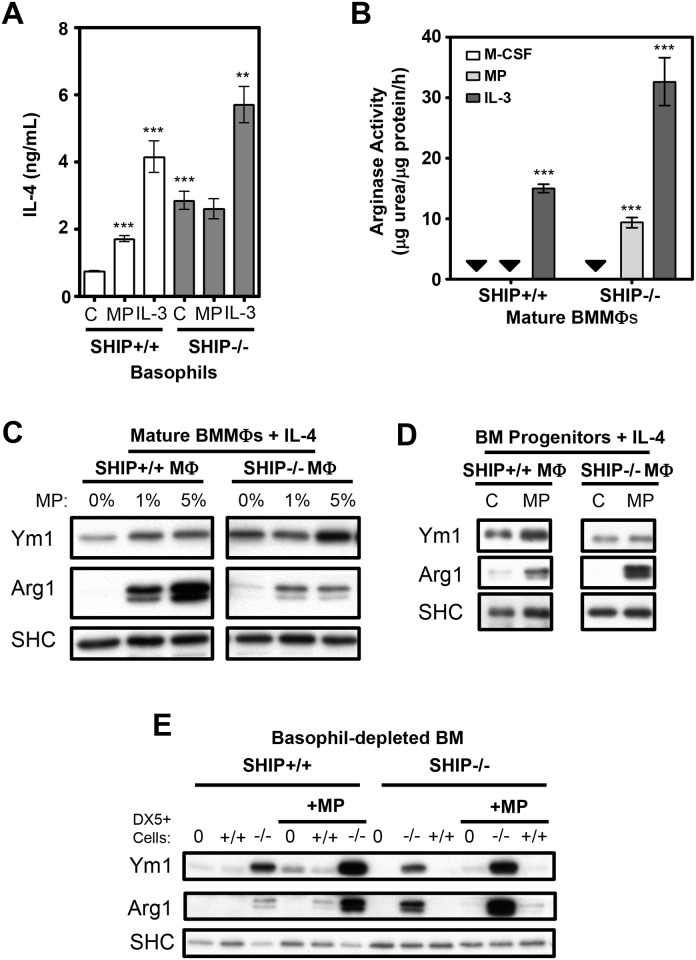
MP increases the sensitivity of macrophages to IL-4-induced M2 skewing. **A)** IL-4 levels secreted by basophils cultured at 5 x 10^5^ cells/mL for 24 h ± 5% MP or 10 ng/mL IL-3. Addition of DX5 Ab does not stimulate IL-4 production from SHIP-/- BM (data not shown). **B)** Arginase activity of mature MФs from the same BM used in panel (A) after 6 days of culture. All results (mean ± SD) are representative of 3 independent experiments. Black arrows indicate levels below detection. **C)** Western blots of mature MФs stimulated for 72 h with IL-4 (0.5 ng/mL) ± the indicated doses of MP. **D)** Western blots of mature MФs derived for 6 days from basophil-depleted BM with M-CSF + IL-4 (1 ng/mL) ± MP (5%). **E)** Western blot of MФs derived from BM for 6 days ± MP (5%) from SHIP+/+ and -/- basophil-depleted (DX5^-^) BM in the bottom wells of a transwell system with basophils (DX5^+^ cells) of varying genotypes co-cultured in a contact-independent manner at a DX5^-^: DX5^+^ ratio of 10:1. 0 = no added DX5^+^ cells. SHC was used as a loading control. All results are representative of 3 independent experiments. ** p < 0.01 compared to relevant control.***p < 0.001 compared to relevant control.

To further address the mechanism through which MP selectively M2-skews SHIP-/- but not SHIP+/+ BM progenitors we first asked if MP was acting on the SHIP-/- basophils to trigger more IL-4 production. Specifically, we stimulated SHIP+/+ and SHIP-/- basophils (ie, DX5+ cells) with MP, and discovered that, unlike IL-3, MP did not increase IL-4 production from SHIP-/- basophils, compared to unstimulated SHIP-/- basophils (Fig 2A). Thus, although MP was causing SHIP-/- MФs to become M2 skewed it was not doing so by increasing IL-4 secretion from SHIP-/- basophils. Importantly, however, SHIP-/- basophils constitutively produced substantially more IL-4 than their SHIP+/+ counterparts (Fig 2A). Also of note, MP significantly increased IL-4 production from SHIP+/+ basophils although the level was still less than SHIP-/- basophils secreted in the absence of stimulation.

Despite the lack of an increase in IL-4 production in MP-stimulated basophils, SHIP-/- BMMФs derived from MP-stimulated BM were M2-skewed while without MP, they were not (Fig 2B). Since MP skewed to an M2 phenotype in an IL-4-dependent manner, but did not do it through increasing IL-4 levels from basophils, we asked if MP was simply increasing the IL-4 sensitivity of MФs. To test this we cultured SHIP+/+ and -/- BM-derived mature MФs (which are adherence selected and thus devoid of basophils and other non-MФ cell types) or BM progenitors with low levels of IL-4 (0.5 ng/ml) in the presence and absence of MP. As shown in Fig 2C and 2D, the addition of MP markedly enhanced the sensitivity of both mature SHIP+/+ and -/- BMMФs and BM progenitors to IL-4-induced M2-skewing.

Importantly, these results suggested that SHIP-/- BM-derived MФs were not more sensitive than SHIP+/+ MФs to IL-4 or to MP-induced sensitization by IL-4. We therefore hypothesized that SHIP-/- but not SHIP+/+ BM becomes M2-skewed by MP because SHIP-/- BM basophils constitutively produce enough IL-4 to M2 skew but only if MP is present to enhance the response to what would otherwise be an insufficient level of IL-4. To test this, we used a transwell system, co-incubating SHIP+/+ and -/- basophils (DX5 + cells) with basophil-depleted (DX5-) BM progenitor cells or mature, pure BMMФs. Confirming our hypothesis, both SHIP+/+ and SHIP-/- BMMФs derived with SHIP-/- basophils were M2-skewed with MP, while those derived either without basophils or with SHIP+/+ basophils failed to robustly M2-skew, even in the presence of MP (Fig 2E). Thus SHIP+/+ and SHIP-/- MФs are not different in their potential to be M2-skewed by IL-4 and the only reason SHIP-/- MФs are M2 skewed while SHIP+/+ MФs are not is the higher constitutive level of IL-4 in the SHIP-/- mouse. Critically, however, this IL-4 cannot M2 skew without MP and so we asked how MP was doing this.

### MP enhances IL-4-induced M2 polarization via ROS and activation of Erk and Akt in both SHIP+/+ and -/- MФs

**Fig 3:**
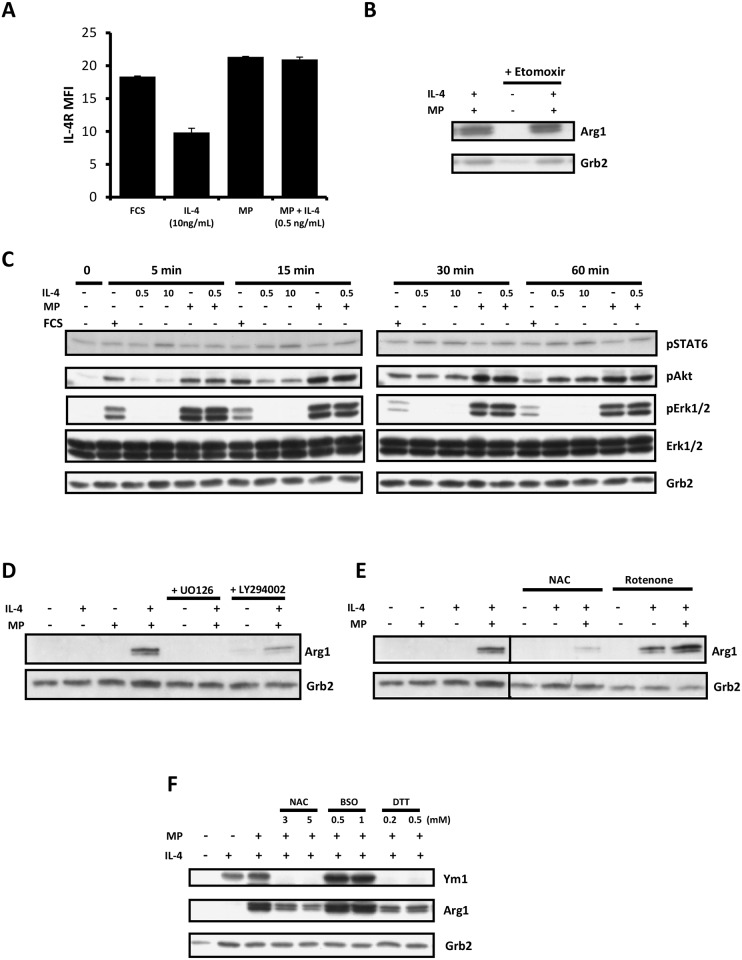
MP enhances IL-4-induced M2 skewing via ROS and activation of Erk and Akt in MФs. **A)** Surface IL-4R expression on WT MФs cultured for 48 h ± 10 ng/mL IL-4, 5% MP, or 5% MP + 0.5 ng/mL IL-4, as assessed by FACS using PE-conjugated anti-IL-4Rα. **B)** Western blot of WT MФs exposed to 5% MP ± etomoxir (100 μM) ± 0.5 ng/mL IL-4 for 72 h and probed for Arg1. Grb2 was used as a loading control. **C)** Western blot of WT MФs treated or not with 10% FCS, 5% MP ± 0.5 ng/mL or 10 ng/mL IL-4 for 5, 15, 30 or 60 min. The blot was probed for pSTAT6, pAKT, pERK1/2 and ERK1/2. Grb2 was used as a loading control. **D)** Western blot of WT MФs exposed for 72 h to the indicated combinations of low dose IL-4 (0.5ng/mL) and 5% MP ± the MEK inhibitor U0126 (5 μM) or the PI3K inhibitor LY294002 (5 μM). **E)** Western blot of WT MФs exposed for 72 h to the indicated combinations of low dose IL-4 (0.5ng/mL) and 5% MP ± N-acetylcysteine (NAC, 2 mM) or rotenone (0.25 μM). **F)** Western blot of WT MФs exposed for 72 h to the indicated combinations of low dose IL-4 (0.5ng/mL) and 5% MP ± low and high doses of N-acetylcysteine (NAC), L-buthionine-S,R-sulfoximine (BSO), or dithiothreitol (DTT). All results are representative of 2 or 3 independent experiments.

To gain some insight into how MP was acting directly on the SHIP+/+ and -/- MФs to enhance IL-4-induced M2 skewing, we first asked if it was simply increasing IL-4 receptor levels on these cells since TGFβ, a factor in MP that we have shown previously plays a role in M2 skewing (Rauh et al), has been reported to increase IL-4Rα levels in mouse microglia [23]. As shown in Fig 3A, this was not the case. After 48 h of incubation, IL-4, at 10 ng/ml, reduced IL-4Rα levels, as expected, but MP or MP + 0.5 ng/ml IL-4 had no effect. Next, since Feng et al showed that M2 polarization is dependent on fatty acid oxidation (FAO) and that the expression of CD36, a receptor for the endocytosis of triacylglycerol-rich lipoprotein particles is induced in MФs by IL-4 [24], we asked if MP-induced M2 skewing involved FAO or increased CD36 expression. As shown in Fig 3B, MP-induced M2 skewing was unaffected by the FAO inhibitor, etomoxir, and CD36 levels were not enhanced (S1 Fig) suggesting MP was not enhancing M2 skewing via increasing FAO.

We then looked at the signaling pathways triggered by FCS, MP, IL-4 and MP + IL-4. We found that IL-4, but not MP or FCS, increased STAT6 phosphorylation, as expected [25] (Fig 3C). On the other hand, MP potently stimulated the phosphorylation of Erk and Akt while FCS (which does not M2 skew) was far less potent, and IL-4 did not trigger Erk phosphorylation at all but did stimulate Akt phosphorylation, albeit with slower kinetics than MP. Adding IL-4 to MP did not increase the phosphorylation of these two signaling proteins. Our finding that FCS was far less effective than MP at activating Erk and Akt suggested that these two signaling proteins might be involved in M2 skewing. To test this we examined M2 skewing by MP in the presence and absence of the MEK inhibitor U0126 and the PI3K inhibitor, LY294002, and found they both dramatically reduced M2 skewing (Fig 3D), at levels that had a negligible effect on viability (data not shown). Similar results were obtained with wortmannin [12]. Since a recent study showed that ROS plays a critical role in the differentiation of human monocytes to M2 skewed macrophages [26] we then asked if M2 skewing could be suppressed by the ROS scavenger, N-acetyl cysteine (NAC) and/or enhanced by the mitochondrial poison, rotenone, which increases ROS [27], and this indeed was the case (Fig 3E). This suggested that mitochondrial derived ROS may play a critical role in MP-induced M2 skewing, perhaps by directly activating Akt [28] and by inactivating the phosphatases that dephosphorylate Erk [29]. Consistent with ROS playing a role we also found the reducing agent dithiothreitol (DTT) inhibited M2 skewing and L-buthionine-S,R-sulfoximine (BSO), the γ glutamyl cysteine synthetase inhibitor (which depletes GSH and thus increases ROS [30], increased M2 skewing (Fig 3F).

### Identification of the factor(s) in MP responsible for enhancing IL-4-induced M2 polarization of MФs

**Fig 4:**
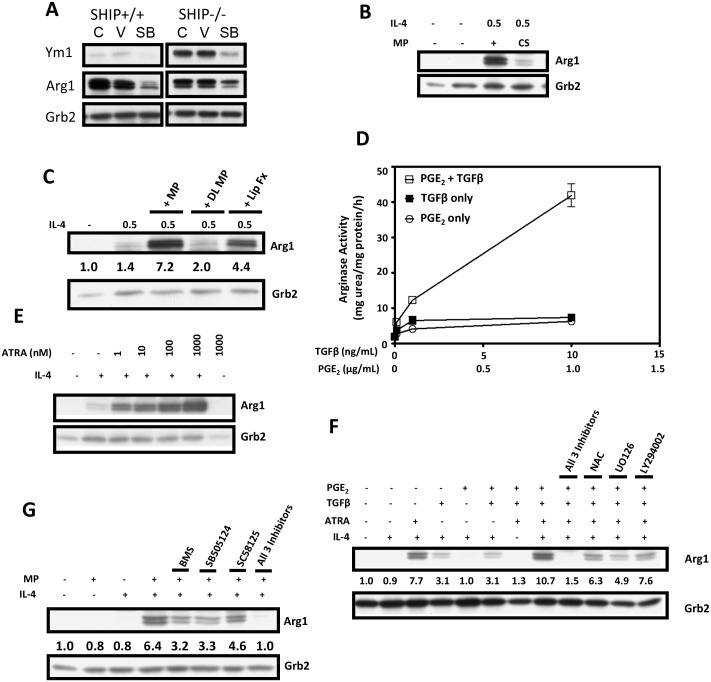
Identification of the factors in MP responsible for enhancing IL-4-induced M2 skewing of MФs. **A)** Western blot of mature SHIP+/+ and -/- MФs stimulated for 72 h with 5% MP + 0.5 ng/mL IL-4 ± nothing (C), vehicle control (DMSO) (V) or 1 μM of the TGFβ inhibitor, SB505124. **B)** Western blot showing the effect of charcoal stripping (CS) on the ability of MP to induce M2 skewing WT MФs in the presence of 0.5 ng/mL IL-4 compared to intact MP. **C)** Western blot showing the effects of MP delipidation (DL MP) and the recovered lipid fraction (Lip Fx) on the ability to M2 skew WT MФs in the presence of 0.5 ng/mL IL-4. **D)** Arginase activity of SHIP-/- BM MФs derived with increasing doses of TGFβ and PGE_2_. All data points are the mean ± SEM of experimental triplicates of 2 independent experiments. **E)** Western blot showing the effect of an ATRA dose response ± 0.5 ng/mL IL-4 on M2 skewing of mature WT MФs after culture for 48 h. **F)** Western blot showing the effect of the combination of 5 μM BMS, 1 μM SB505124 and 20 μM SC28125 as well as 3 mM NAC, 5 μM UO126, and 5 μM LY294002 on ATRA (4 nM) + TGFβ (2 ng/mL) + PGE_2_ (3 ng/mL) induced M2 skewing of WT MФs ± 0.5 ng/mL IL-4 after 48 h in culture. **G)** Western blot showing the effect of BMS SB505124 and SC58125 at the same concentrations as in (F) and the combination of all three on MP-induced M2 skewing of WT MФs ± 0.5 ng/ml IL-4 after 48 hr in culture. For panels C, F and G, densitometry was performed and expression levels of Arg1, relative to Grb2 levels, are indicated. All results are representative of 2 or 3 independent experiments.

In earlier studies we established, using bead-bound antibodies to TGFβ and IL-10 to deplete them from MP, that TGFβ but not IL-10, was involved in MP-induced M2-skewing [12]. To pursue this further we tested the TGFβ pathway-specific inhibitor, SB-505124, (which inhibits the TGFβ type 1 receptor activin receptor-like kinases (ALK) 4,5 and 7 [31]), and found that it significantly reduced MP plus IL-4-induced M2-skewing of both SHIP+/+ and -/- MФs (Fig 4A). Since this only partially blocked M2-induced skewing we attempted several fractionation procedures with MP to identify other factors that might be involved and found that charcoal stripping markedly reduced the M2 skewing ability of MP (Fig 4B). This suggested that a lipid soluble factor might be involved and we pursued this further by performing a gentle lipid extraction which retains proteins in the aqueous phase in their native conformation [18] and found that, indeed, substantial M2 skewing activity was present in the lipid fraction (Fig 4C). Since the lipid soluble prostaglandin, PGE_2_, has been reported to be involved in M2 skewing [32,33] we then tested it with and without TGFβ. As shown in Fig 4D, TGFβ plus PGE_2_ acted synergistically to enhance M2 skewing but even at levels substantially higher than that observed physiologically [34,35], they were far less potent than 5% MP at M2 skewing.

Since the lipid soluble vitamin A metabolite, ATRA, has also recently been reported to play a role in M2 skewing [36], we then tested it in lieu of MP for its M2 skewing ability and found that it was indeed a potent M2 skewing factor, triggering substantial Arg1 expression at concentrations as low as 1 nM. Importantly, like MP, it had no M2 skewing ability in the absence of low levels of IL-4 (Fig 4E).

We then tested the combination of ATRA, TGFβ and PGE_2_, at levels reported to be present in MP [34,35] for their ability to M2 skew and found that, together, they elicited Arg1 levels similar to that of 5% MP (Fig 4F). Also, the ability of the combination of ATRA, TGFβ and PGE_2_ to M2 skew was markedly inhibited by the inhibitors, NAC, U0126 and LY294002 (Fig 4F), mimicking the response of MP to these agents. As well, using specific inhibitors of each of them, i.e., BMS195614 (BMS) for ATRA (a selective RARα antagonist) [37], SB-505124 for TGFβ and the COX2 inhibitor, SC-58125, for PGE_2_ we found that, as expected, they totally eradicated M2 skewing.

Lastly, to determine if ATRA, TGFβ, and PGE_2_ actually participated in MP-induced M2 skewing, we tested BMS, SB-505124 and SC-58125, at the same concentrations used to treat ATRA, TGFβ and PGE_2_, to see if they reduced the M2 skewing capacity of MP. As shown in Fig 4G, each inhibitor alone significantly reduced the M2 skewing capacity of MP but, more importantly, addition of all 3 inhibitors reduced M2 skewing of MP to baseline levels achieved with low levels of IL-4 alone.

## Discussion

In order to evaluate, in vitro, potential therapeutic agents for their efficacy in skewing MФs to an M1 or M2 phenotype it is important to use culture conditions that mimic, as closely as possible, in vivo conditions. Our studies strongly suggest that the standard culture conditions used for both the derivation of murine MФs from BM and the skewing of mature MФs (i.e. 10% FCS + 5–10 ng/ml M-CSF) do not reflect the in vivo environment and that the addition of 5% MP brings it more into line with what is happening *in vivo*, at least with regard to the M1 and M2 skewing of SHIP+/+ and -/- BM MФs. Specifically, the addition of 5% MP not only increases the expression of Arg1 and Ym1 but TGFβ and IL-10 as well and MP also leads to the reduced synthesis of M1 markers (TNFα, CCL3 and NO) in response to LPS [12]. Related to this, we found that, in addition to MP, both human serum and human plasma M2 skew as well. Also, unlike FCS, calf serum M2 skews, suggesting that fetal serum lacks factors that promote (or possess factors that inhibit) M2 skewing. Using these new culture conditions has allowed us to elucidate, to some extent, the factors responsible for the M2 skewing of both WT and SHIP-/- BM MФs under homeostatic (i.e. uninfected) conditions in the mouse. Using both 6 day assay cultures, with SHIP+/+ and -/- murine BM depleted of mature MФs (adherent cells) to see the effects of basophils, IL-4, etc during differentiation, and mature BM-derived MФs to look at factors that affect the skewing of mature MФs toward an M1 or M2 phenotype, we have obtained evidence that MP pushes both towards an M2 phenotype if a low but necessary level of IL-4 is present to support this skewing.

**Fig 5:**
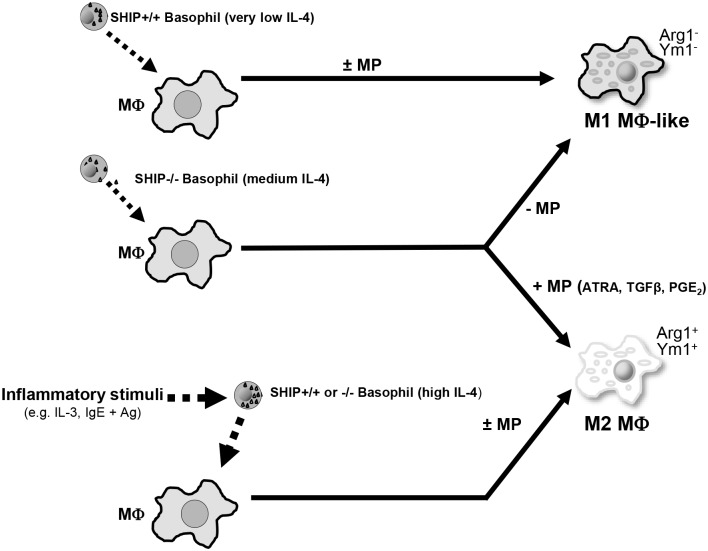
Proposed model of M2-skewing of SHIP+/+ and -/- MФs. SHIP-/-, but not +/+, basophils constitutively secrete sufficient IL-4 to M2 skew SHIP+/+ or -/- progenitors or mature MФs but only in the presence of ATRA, TGFβ and PGE_2_ in MP. These MP factors synergize with low levels of IL-4 to M2 skew the MФs under non-inflamed conditions.

Specifically, our results suggest a model for M2 MФ skewing (see Fig 5) in which basophils play a central role. Basophils that lack SHIP constitutively secrete more IL-4 than WT basophils as one might expect given that IL-4 production has been shown to be PI3K dependent [13,14]. In the presence of MP there is approximately 2 to 10 fold (data not shown) more IL-4 secreted/cell from SHIP-/- than SHIP+/+ basophils. Compounding this difference, SHIP-/- mice suffer from basophilia and have 2–3 fold more basophils in their BM and peripheral blood [13]. However, this level of IL-4 is still insufficient to skew MФs to an M2 phenotype in the absence of MP. Importantly, while SHIP-/- basophils produce more IL-4 than WT basophils, our transwell experiments highlight the ability of SHIP+/+ basophils to M2-skew MФ to M2-ske and the only reason WT MФs are not M2 skewed during differentiation is because WT basophils produce less IL-4 than SHIP-/- basophils and that if adequate IL-4 is provided, both WT and SHIP-/- MФs will be M2 skewed to the same extent when incubated with MP. Of note, in these transwell studies, SHIP-/- basophils alone were capable of M2-skewing MФs, suggesting that, given sufficient numbers, basophils through their PI3K activation status and trafficking, are likely important mediators of M2 skewing in general and not just in the perturbed, SHIP-/- mouse.

To elucidate how MP promotes M2 skewing we carried out mechanism of action studies and found that MP was not promoting M2 skewing by further activating basophils but rather by acting directly on MФ but rather by heir responsiveness to IL-4. This increased responsiveness does not appear to be mediated via either increased cell surface IL-4Rs on MФ (in contrast to recent studies showing that TGFβ enhances M2-like skewing of murine microglia by upregulating IL-4Rα levels [23]) or by increasing FAO (in contrast to recent elegant studies from the Pearce lab showing that M2 MФ polarization is dependent on FAO and is accompanied by an increase in cell surface expression of CD36, a receptor involved in taking up triacylglycerol-rich lipoprotein particles [24]). On the contrary, our results, using NAC, DTT, rotenone, BSO, U0126 and LY294002 are consistent with MP enhancing IL-4-induced M2-skewing via mitochondrial ROS production. This is interesting since ROS have traditionally been associated with M1 MФ BSO, U0126 and LY294002gest that they play a role in M2 polarization as well [26,39]. ROS, in turn, is capable of activating Akt [28] and preventing the deactivation of the Erk pathway (via ROS-induced inactivation of deactivating phosphatases [29,40]). However, a caveat to this model is that, after carrying out many experiments with both dihydroethidium (DHE) and 2’,7’–dichlorofluorescin diacetate (DCFDA) to stain ROS, we only obtained a non-statistically significant trend towards increased ROS in MФ we only obtained a non-statistically significant trend tdetected ROS levels. This may have been due to rapid reduction of ROS to maintain Erk phosphorylation.

Our studies to characterize the specific factors in MP that are responsible for increasing the sensitivity of MФs suggest that TGFβ, which we have shown previously to play a role in MP-induced M2 skewing [12], collaborates with ATRA and PGE_2_ in MP to sensitize MФs to IL-4-induced M2 skewing. Our findings with ATRA are in agreement with a recent report showing that ATRA, while having no effect on its own, dramatically potentiates IL-4-induced Arg 1 in BM-derived mouse MФs [36]. Also of note, since PGE_2_ has a half life in plasma of only 30 sec, the inhibition of MP-induced M2 skewing that we observe with the COX2 inhibitors SC58125 (Fig 4F) and celecoxib (data not shown), are likely due to blocking of PGE_2_ production and its autocrine effects on our MФs. Related to this, both ATRA and TGFβ, and likely other factors in MP, appear to stimulate the production of PGE_2_ [41,42].

Both mice and humans have been shown to display a wide spectrum of M1 to M2 skewing under unchallenged (homeostatic) conditions and this, in turn, has been shown to have a profound influence on their ability to fight off infections and prevent various disorders [43,44]. Understanding all the factors involved in skewing is a key first step towards being able to manipulate this skewing to reduce the severity of these diseases. For example, while PGE_2_ has both pro and anti-inflammatory properties, many tumours secrete PGE_2_ to promote their own growth by skewing the immune system to a “healing” M2 phenotype and we as well as many others have shown that you can prevent as well as slow down the growth of many tumours with COX2 inhibitors [45]. As well, since atherosclerosis is promoted by M1 macrophages [46] and asthma and allergies are worsened by M2 skewed macrophages, reducing ATRA, PGE_2_ and/or TGFβ may prove efficacious in reducing the symptoms of allergies, asthma and the incidence and progression of cancer while increasing these factors might reduce certain forms of heart disease.

While many molecules have been reported to be capable of M1 and M2 skewing [43,44], there is very little evidence to date on which factors in plasma actually play a role. Interestingly, ATRA, TGFβ and PGE_2_ are distinct from the well-established M2-skewing factors known to be involved during inflammation (e.g. IL-3, IgE + Ag). Based on our results we hypothesize that under homeostatic, unchallenged conditions where IL-3 and IgE + Ag are not major players in MФ polarization, ATRA, TGFβ and PGE_2_ likely play a significant role in M2 skewing. This is supported by our IL-3 neutralization experiments demonstrating that IL-3 itself does not play a role in MP-mediated M2-skewing under homeostatic conditions. However, it is likely that many other factors, such as IL-6 [32], IgG immune complexes [47], etc, also play a role. Under inflammatory conditions where IL-3 levels are elevated or when allergen inducing IgE triggering occurs on basophils, sufficient IL-4 is secreted from both SHIP+/+ and -/- basophils to trigger M2 MФ skewing in the absence of these MP factors.

With regard to the regulation of ATRA, TGFβ and PGE_2_ levels in human plasma, ATRA levels have been shown to be impacted by both diet [48] and genetics (via the enzymes that generate it from vitamin A (retinol) and degrade it [49]). TGFβ levels, on the other hand, are regulated in large part by release from inhibitory binding proteins in tissues and plasma and from activated platelets. Also, it, as well as PGE_2_, is often secreted by human tumor cells to repress anti-tumor immunity [50,51].

In summary, our studies suggest that ATRA, PGE_2_ and TGFβ within MP all play a significant role in M2 skewing *in vivo* and may serve as an additional microenvironmental stimulus for M2 skewing when IL-4 levels are low. This makes them, together with SHIP [38], attractive targets for either the induction or suppression of M2-polarization, depending on the disorder to be treated.

## Supporting Information

S1 Fig Cell surface CD36 levels are not enhanced on MФs in response to MP. Surface CD36 expression on SHIP+/+ MФs cultured for 48 h ± 10 ng/mL IL-4, 5% MP, or 5% MP + 0.5 ng/mL IL-4, as assessed by FACS.
(TIF)
