# Supplementary figures and images for: All Trans Retinoic Acid, Transforming Growth Factor β and Prostaglandin E2 in Mouse Plasma Synergize with Basophil-Secreted Interleukin-4 to M2 Polarize Murine Macrophages
Source: PLoS One. 2016 Dec 15;11(12):e0168072. doi: 10.1371/journal.pone.0168072 (PMC5158015; doi:10.1371/journal.pone.0168072)

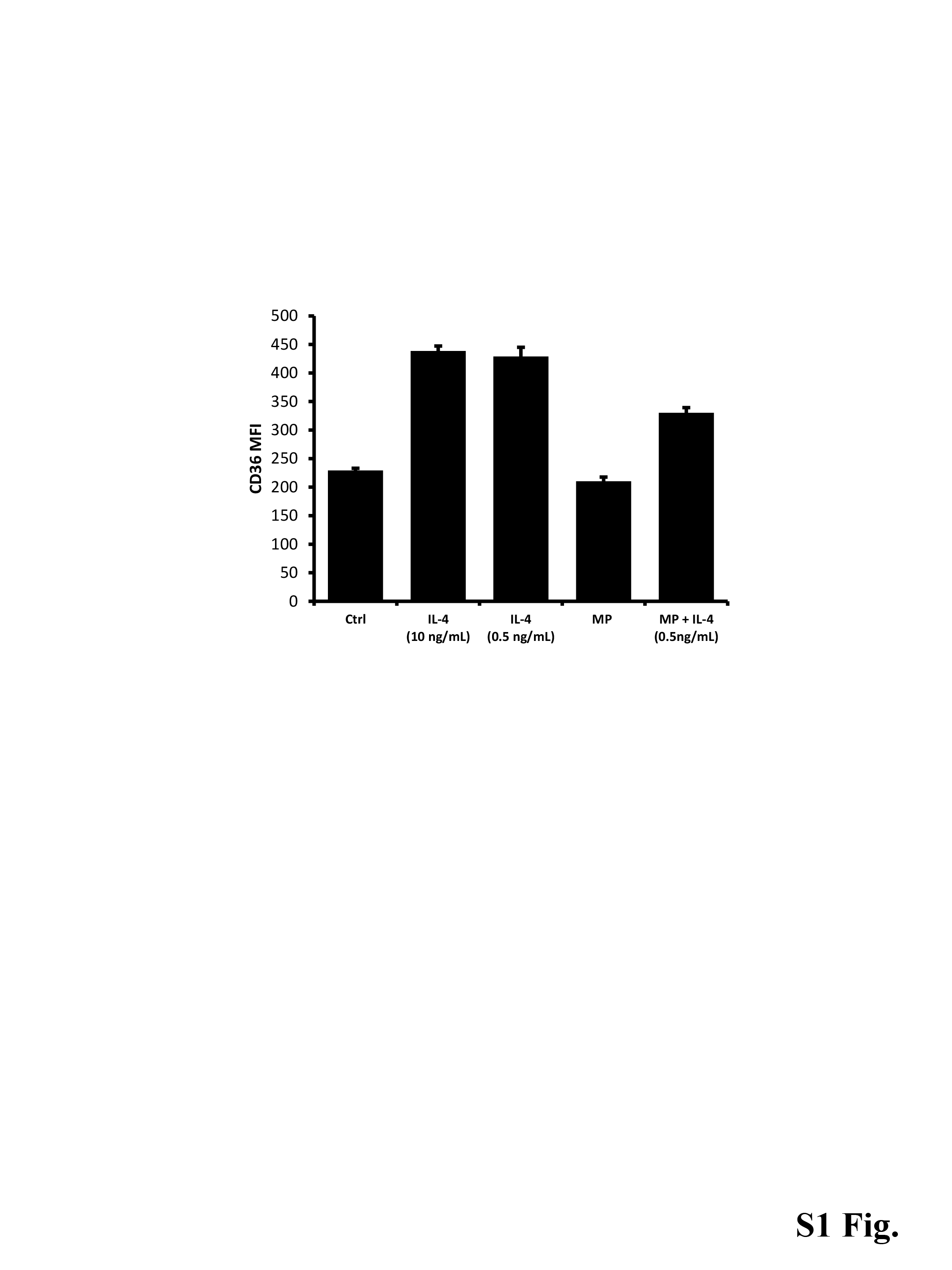

Supplement: S1 Fig — Surface CD36 expression on SHIP+/+ MФs cultured for 48 h ± 10 ng/mL IL-4, 5% MP, or 5% MP + 0.5 ng/mL IL-4, as assessed by FACS. (TIF) [file pone.0168072.s001.tif]
